# Enhanced efficacy in drug-resistant cancer cells through synergistic nanoparticle mediated delivery of cisplatin and decitabine†

**DOI:** 10.1039/c9na00684b

**Published:** 2020-01-27

**Authors:** M. Parhizkar, P. J. T. Reardon, A. H. Harker, R. J. Browning, E. Stride, R. B. Pedley, J. C. Knowles, M. Edirisinghe

**Affiliations:** School of Pharmacy, University College London London UK maryam.parhizkar@ucl.ac.uk; Mechanical Engineering, University College London London UK; Division of Biomaterials and Tissue Engineering, UCL Eastman Dental Institute, University College London London UK; Department of Physics and Astronomy, University College London London UK; Institute of Biomedical Engineering, Department of Engineering Science, University of Oxford Oxford UK; UCL Cancer Institute, Department of Oncology, University College London London UK; The Discoveries Centre for Regenerative and Precision Medicine UCL Campus London UK; Department of Nanobiomedical Science, BK21 PLUS NBM Global Research Center for Regenerative Medicine, Dankook University Cheonan 31114 Republic of Korea; UCL Eastman-Korea Dental Medicine Innovation Centre, Dankook University Cheonan 31114 Republic of Korea

## Abstract

There are several limitations with monodrug cancer therapy, including poor bioavailability, rapid clearance and drug resistance. Combination therapy addresses these by exploiting synergism between different drugs against cancer cells. In particular, the combination of epigenetic therapies with conventional chemotherapeutic agents can improve the initial tumour response and overcome acquired drug resistance. Co-encapsulation of multiple therapeutic agents into a single polymeric nanoparticle is one of the many approaches taken to enhance therapeutic effect and improve the pharmacokinetic profile. In this study, different types of poly(lactic-*co*-glycolic acid) (PLGA) nanoparticles (NPs), matrix and core–shell (CS), were investigated for simultaneous encapsulation of a demethylating drug, decitabine, and a potent anticancer agent, cisplatin. It was shown that by altering the configuration of the CS structure, the release profile could be tuned. In order to investigate whether this could enhance the anticancer effect compared to cisplatin, human ovarian carcinoma cell line (A2780) and its cisplatin resistant variant (A2780cis) were exposed to free cisplatin and the CS–NPs. A better response was obtained in both cell lines (11% and 51% viability of A2780 and A2780cis, respectively) using CS–NPs than cisplatin alone (27%, 82% viability of A2780 and A2780cis, respectively) or in combination with decitabine (22%, 96% viability of A2780 and A2780cis, respectively) at equivalent doses (10 μM).

## Abstract 2

Co-encapsulation of cisplatin and decitabine in nanoparticles produced by electrohydrodynamic processing. Combination of multiple therapeutic agents in a single nano-carrier could potentially help to overcome drawbacks of monotherapy in cancer treatment.

## Graphical abstract

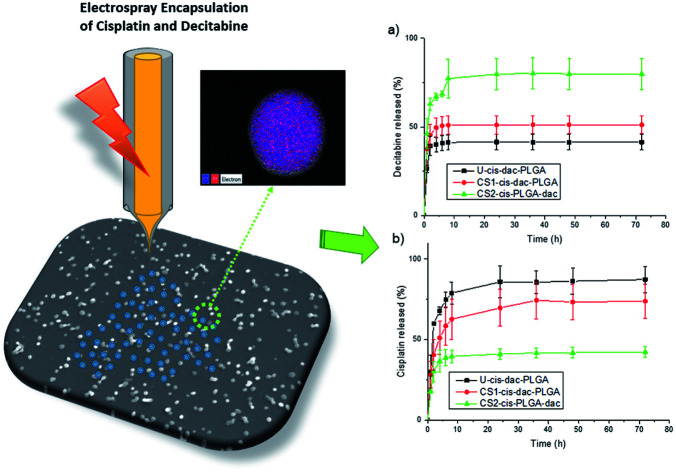

## Introduction

Treatment of multifunctional diseases such as cancer typically cannot be achieved by therapeutic agents that inhibit a single target.^1^ Single agent therapeutics often lead to development of drug resistance as well as limited accessibility of drug to tumour tissue due to intra-tumour heterogeneity.^2^ Moreover, in order to achieve therapeutically relevant concentrations within the tumour, high systemic doses of chemotherapy agents need to be administered, often resulting in severe toxic side effects.^3^ This is the case with drugs such as cisplatin and other platinum derivative drugs that are widely used in the treatment of ovarian cancer and head and neck squamous cell carcinoma (HNSCC).^4,5^ The primary mechanism of action of cisplatin is through DNA damage.^6^ However, several cellular pathways are activated by cisplatin exposure, including DNA repair pathways that remove the damage and result in the emergence of drug resistance.^7^ It has been shown that a combination of multiple anticancer drugs can prevent this resistance through administration of lower doses of the individual drug.^8,9^ More recent studies have proposed combining conventional chemotherapy with ribonucleic acid inhibitors (RNAi) and demethylating drugs.^10–12^ Viet *et al.* showed that decitabine, a demethylating drug, reversed methylation and gene expression toward a cisplatin-sensitive profile in treatment of cisplatin resistant HNSCC cells.^10^ Steele *et al.* have similarly shown that treatment of ovarian and colon cell lines with the decitabine results in partial reversal of DNA methylation and sensitisation to cisplatin and carboplatin both *in vitro* and *in vivo*.^13^

Combination therapy has already been shown to be clinically effective as a result of drug synergy effects, reduced toxicity and suppression of multi-drug resistance (MDR) through different mechanisms of action.^14^ However, current combination therapies are sub-optimal due to the differing pharmacokinetic profiles and biodistribution of different drug molecules.^15,16^ In recent years, many approaches have been explored to deliver multiple therapeutic agents in a single nanocarrier^17,18^ and shown potential for the treatment of various types of cancer.^19,20^ Polymeric nanoparticles (NPs) with compartmentalised structures, in particular, have emerged as an effective strategy for successful delivery of anticancer drugs.^21,22^ NPs are promising candidates for combination therapy due to their high surface area to volume ratio, ready separation of incompatible drugs into physically distinct environments and the ability to tune drug release rates *via* the use of degradable polymers.^23^ Nevertheless, producing complex NPs *via* conventional methods such as precipitation, oil-in-water (O/W) single emulsion, and water-in-oil-in-water (W/O/W) double emulsification^24,25^ poses a significant challenge. Co-axial and multi-needle electrohydrodynamic atomisation (EHDA) technology has been shown to offer an alternative method for generating nanoparticulate compartmentalised systems with several advantages^26^ including high encapsulation efficiency and a high degree of control over particle size and distribution.^27^ Other significant advantages of drug encapsulation using EHDA include the reproducibility and speed with which NP can be generated; the fact that it is a single step process that does not require removal of solvents or templating agents through further procedures such as lyophilisation that lead to morphological and structural changes.^28,29^

The aims of this study are to investigate the feasibility of encapsulating two drugs (cisplatin and decitabine) in a single nano carrier using EHDA; to control the release rates of both drugs by varying the internal structure of NPs from a single matrix to a core–shell (CS) particle; and finally, to investigate their anticancer effect in normal and cisplatin resistant cell lines.

## Methods

### Materials

Poly(lactic-*co*-glycolic acid), PLGA, (copolymer 50 : 50, Resomer RG503H, molecular weight of 33 000 Da, inherent viscosity 0.41 dl g^−1^) was supplied from Boehringer Ingelheim (Ingelheim, Germany). Dimethylacetamide (DMAc) was purchased from Sigma Aldrich (Poole, UK). Decitabine was purchased from Cell Guidance Systems (Cambridge, UK). Cisplatin (*cis*-Platinum(ii)diamine dichloride, molecular weight of 300 g mol^−1^) was obtained from Enzo Life Sciences (Exeter, UK).

### Particle fabrication

In order to prepare the single matrix “uniform” particles (U-cis-dac-PLGA) using single needle EHDA, PLGA solutions (2 wt%) were prepared by dissolving the polymer in DMAc and mechanically stirring for 400 s. Cisplatin (2 mg mL^−1^) and decitabine (2 mg mL^−1^) were added to the solution followed by stirring for a further 500 s in ambient temperature (20 °C) to ensure the total dissolution of the drugs and polymer. To form the CS (CS) particles, a coaxial EHDA system was used and 4 different solutions were prepared. For CS particles with both drugs in the core (CS1-cis-dac-PLGA), the same amount of cisplatin (2 mg mL^−1^) and decitabine (2 mg mL^−1^) were dissolved in DMAc, while 2 wt% PLGA was dissolved in the same solvent (DMAc) to prepare the inner and outer solutions, respectively. For CS particles with cisplatin in the core and decitabine in the shell (CS2-cis-PLGA-dac), the same amount of cisplatin (2 mg mL^−1^) was dissolved in DMAc, while decitabine (2 mg mL^−1^) and 2 wt% PLGA was dissolved in the same solvent (DMAc) to prepare the inner and outer solutions, respectively.

**Fig. 1:**
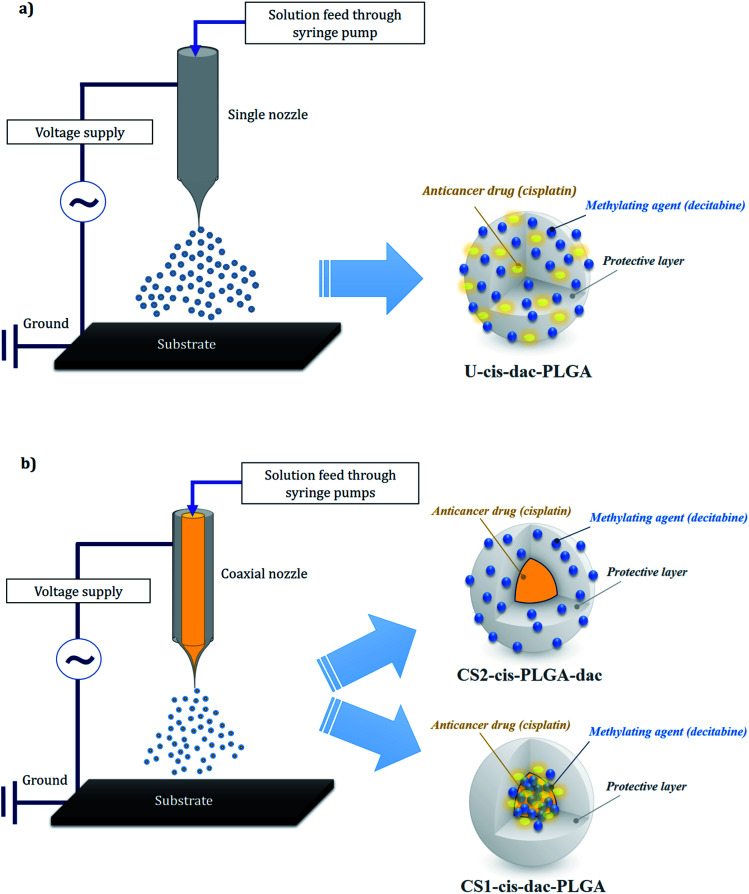
Single (a) and coaxial (b) electrospraying configurations and corresponding drug distribution within the particle structure.

The solutions were processed using both single needle and coaxial EHDA setups (Fig. 1). For the single needle configuration, the solutions were made to flow through a stainless steel needle (18G, ID: 0.84 mm and OD: 1.27 mm) using a syringe pump (PHD 4400, Harvard Apparatus Limited, Edenbridge, UK) at a constant flow rate of 3 μL min^−1^. In the coaxial configuration, the inner and outer solutions were fed through coaxial stainless steel needles of 19G (ID: 0.69 mm and OD: 1.07 mm) and 16G, (ID: 1.2 mm and OD: 1.6 mm), respectively. In order to achieve a stable cone jet, the flow rate for these solutions was 1 μL min^−1^ for both inner and outer solutions.

An electric potential difference between the needle and a ground electrode to the solution was applied through a high precision voltage generator (Glassman Europe Ltd, Bramley, UK). The applied voltage was varied from 17 to 20 kV as required to form a stable cone jet. A working distance of 200 mm below the device exit was adjusted to collect the dried particles directly onto glass slides or a stainless steel substrate respectively for characterization and measurement of drug release. A Leica DMS300 camera was used to monitor the jet and particle formation processes. Experiments were conducted at the ambient temperature of 19–21 °C and relative humidity of 40–50%. Experiments were conducted at least 5 times to ensure the reproducibility of the EHDA process and consistency of the particles produced.

### Particle characterization

#### Optical microscopy and scanning electron microscopy

In the first instance, particle samples collected on glass slides were analysed under an optical microscope (Nikon Eclipse ME 600) fitted with a camera (Micropublisher 3.3 RTV, 3.3 megapixel CCD Color-Bayer Mosaic, Real Time Viewing camera, Media Cybernetics, Marlow, UK). The particle architecture and morphology were then examined using scanning electron microscope (SEM, XL30 FEG, Philips). Both optical and scanning electron micrographs were analysed to obtain the average diameter and standard deviation of the population of particles by randomly measuring 300 particles from each sample (Image J software). The presence of cisplatin in the NPs was confirmed by an INCA X-sight EDAX system (Oxford Instruments) with the XL30 microscope for EDX (Energy dispersive X-ray) spectroscopy analysis.

#### Dynamic light scattering (DLS)

The NPs were collected in water and immediately tested using DLS. Intensity mean hydrodynamic size of the nanoparticles were measured on a Malvern Zetasizer Ultra (Malvern Instruments, Malvern, UK) incorporating Non-Invasive Back Scatter (NIBS) and, uniquely, Multi-Angle Dynamic Light Scattering (MADLS). The measurements were carried out at 25 °C and scattering angles of 173, 13 and 90° for NIBS, forward and side scatter, respectively, and water was used as the dispersion medium.

#### Transmission electron microscopy

In order to further study the structural characteristics and cisplatin distribution within the NP formulations, particles were electrosprayed directly onto carbon coated copper grids. The samples were examined without additional contrast using a transmission electron microscope (TEM, CM12, Philips), and EDX analysis (JEM-2100-Jeol TEM fitted with X-max EDAX system-Oxford Instruments).

#### Fourier transform infrared spectroscopy

The infrared spectra of cisplatin, decitabine, PLGA NPs and drug loaded NPs were recorded using a Fourier Transform Infrared (FTIR-ATR-Perkin Elmer 2000) spectrophotometer. IR Spectra of all materials were recorded using a frequency range of 400–4000 cm^−1^, and averaged over 4 runs. Powdered samples were placed on the Attenuated Total Reflectance (ATR) crystal, and then compressed using an axial screw.

### *In vitro* drug release

Following a previously published protocol, 20 mg of cisplatin and decitabine loaded NPs were dispersed in 1.5 mL of PBS (pH 7.4) and incubated at 37 °C. At predetermined time intervals, 0.5 mL aliquots of solution were removed for the purpose of measurement, and replaced with fresh buffer solution. Aliquots of the supernatant were centrifuged and analysed using High Performance Liquid Chromatography (HPLC). Liquid chromatography was optimised on a Hypercarb 5 μM 250 × 4.6 mm HT column (Thermo Scientific) under isocratic conditions. A mobile phase of NaCl soln. (0.9% w/v) and acetonitrile (90 : 10) was used at a flow rate of 1 mL min^−1^. In order to determine the encapsulation efficiency of cisplatin, 10 mg of cisplatin-loaded nano/micro particles was mixed with DMAc followed by addition of PBS. The solution was then passed through a 0.22 μm filter and analysed by HPLC. Encapsulation efficiency (percentage of the amount of drug added initially that was entrapped in the NPs) was calculated using below formula:

1 Encapsulation efficiency (EE%) = (*W*_t_/*W*_i_) × 100%

where *W*_t_ is the actual drug loading and *W*_i_ is the weight of drug used in particle synthesis.

### Analytical model for the drug release

In order to allow quantitative comparison, mechanistic mathematical models (NonlinearModelFit function within Mathematica (Wolfram Research, Champaign, IL, USA)) were applied to the cisplatin and decitabine release profiles. Quality of fit was assessed using the adjusted *R*^2^ parameter (the adjustment allows for different numbers of fitting parameters). The expressions for release rates were given for the situations in which material is contained only within a core region or a shell of a spherical particle. Although the solubility of the drugs used in the experiments are quite low, the experimental results do not suggest that the release rate is strongly affected by the sampling rate, therefore perfect sink boundary conditions were adopted rather than including a partition coefficient between the particle and the surrounding fluid. For ease of fitting release rates, simple analytical fits were applied. Finally, the observed release rates were interpreted in terms of diffusion coefficients. A simple diffusion model, with diffusion coefficients independent of time was adopted. On the time-scale of the experiments significant degradation of the PLGA matrix would not be expected.

#### Analytical method

First particles are considered in which a drug is initially uniformly distributed, and diffuses with diffusion coefficient *D* out of a sphere of radius *a*. It is convenient to work with a scaled time *τ* = *tD*/*a*^2^. For perfect sink boundary conditions, standard results according to which the fractional release from a sphere is used.^30^2
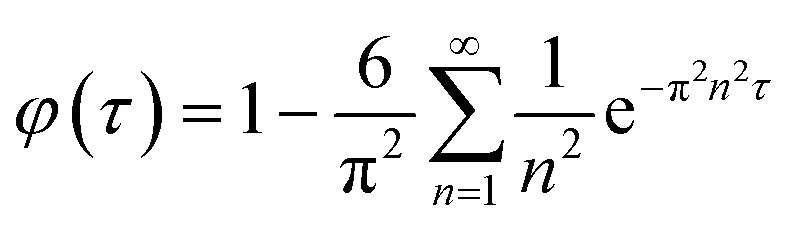


Assuming that the diffusion coefficient is constant throughout the CS particle, if the initial drug concentration is constant in the region *r* < *ρa* and zero otherwise the fractional release rate is:3
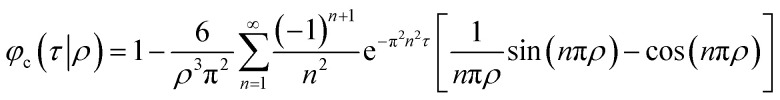


Whilst, if the initial drug concentration is constant in the region *ρa* < *r* < *a* and zero otherwise the fractional release rate is:4
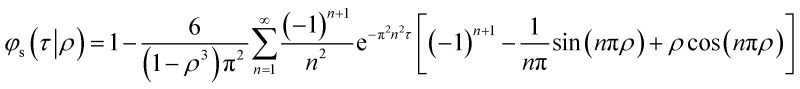


As expected, eqn (3) reduces to eqn (2) for *ρ* = 1, as does eqn (4) for *ρ* = 0.

#### Analytical approximation

As it is demanding to fit the experimental data in the infinite series developed in the previous section, an analytical expression was used in this context. In previous work,^31^ an approximation for *φ* to in eqn (2) was suggested:5
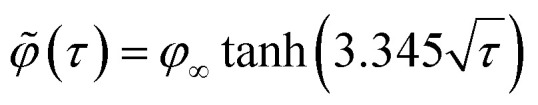
Here, *φ*_∞_ is the limiting release fraction observed after a long time. Finding a satisfactory expression to fit *φ*_c_ or *φ*_s_ as functions of both *τ* and *ρ* is difficult. As a result, we developed fits for a specific value of *ρ*, namely *ρ* = 0.8, corresponding to equal volumes of material in the core and in the shell. The results are:

6 *

<svg xmlns="http://www.w3.org/2000/svg" version="1.0" width="10.842105pt" height="16.000000pt" viewBox="0 0 10.842105 16.000000" preserveAspectRatio="xMidYMid meet"><metadata>
Created by potrace 1.16, written by Peter Selinger 2001-2019
</metadata><g transform="translate(1.000000,15.000000) scale(0.009211,-0.009211)" fill="currentColor" stroke="none"><path d="M320 1480 l0 -40 -40 0 -40 0 0 -80 0 -80 40 0 40 0 0 40 0 40 40 0 40 0 0 40 0 40 40 0 40 0 0 -40 0 -40 40 0 40 0 0 -40 0 -40 120 0 120 0 0 40 0 40 40 0 40 0 0 80 0 80 -40 0 -40 0 0 -40 0 -40 -40 0 -40 0 0 -40 0 -40 -40 0 -40 0 0 40 0 40 -40 0 -40 0 0 40 0 40 -120 0 -120 0 0 -40z M320 1080 l0 -40 -40 0 -40 0 0 -40 0 -40 -40 0 -40 0 0 -80 0 -80 -40 0 -40 0 0 -200 0 -200 40 0 40 0 0 -40 0 -40 80 0 80 0 0 -160 0 -160 40 0 40 0 0 120 0 120 40 0 40 0 0 80 0 80 120 0 120 0 0 40 0 40 40 0 40 0 0 40 0 40 40 0 40 0 0 240 0 240 -40 0 -40 0 0 40 0 40 -120 0 -120 0 0 -40 0 -40 -40 0 -40 0 0 -160 0 -160 -40 0 -40 0 0 -120 0 -120 -80 0 -80 0 0 160 0 160 40 0 40 0 0 80 0 80 40 0 40 0 0 80 0 80 -40 0 -40 0 0 -40z m480 -200 l0 -80 -40 0 -40 0 0 -80 0 -80 -40 0 -40 0 0 -80 0 -80 -80 0 -80 0 0 80 0 80 40 0 40 0 0 160 0 160 120 0 120 0 0 -80z"/></g></svg>

*_c_ (*τ*|0.8) = *φ*_∞_ tanh(5.3928*τ*^0.8746^)

7 **_s_ (*τ*|0.8) = *φ*_∞_ tanh(4.0764*τ*^0.3969^)

Experimental results were then analysed using these models (ESI, Fig. A1 and A2†).

### *In vitro* cell cytotoxicity studies

Cell viability was determined in a human ovarian carcinoma cell line (A2780) and its cisplatin resistant variant (A2780cis) obtained from Sigma. Cells were grown in (RPMI)-1640 medium supplemented with 10% fetal bovine serum (FBS). For the 3-(4,5-dimethylthiazol-2-yl)-2,5-diphenyltetrazolium bromide (MTT) assay (Sigma-Aldrich, St. Louis, MO, USA), cultured A2780 and A2780cis cells were seeded into 96-well flat-bottomed plates at a density of 5 × 10^3^ cells in 100 μL of medium and incubated for 24 h in a 5% CO_2_ atmosphere at 37 °C. They were then incubated in growth medium containing different concentrations of decitabine, cisplatin or equivalent cisplatin/decitabine-loaded CS2-cis-PLGA-dac NPs for 72 h. Media containing different cisplatin dosages were made up from successive dilutions in warmed media, from a stock solution of cisplatin in sterile PBS (1 mM). After treatment, MTT (5 mg mL^−1^ in PBS) was diluted 1 : 100 with medium into each well. After 2 h of incubation, culture supernatants were aspirated, and purple insoluble MTT product was dissolved in 100 μL of dimethyl sulfoxide (DMSO)/ethanol (EtOH) (50 : 50) for 10 min. The absorbance in each well was recorded at 570 nm using a microplate reader; blanks were subtracted from all data, and the results were analysed using Origin software (OriginLab, Northampton, MA, USA).

## Results

### Nanoparticles characteristics

Previously, we have utilised EHDA as an effective technique for encapsulating cisplatin, with high loading efficiency (>70%), into PLGA NPs using both single needle and coaxial configurations.^32,33^ We have shown that the particle architecture plays an important role in the distribution of the drug and consequently the drug release profile. The CS structure of particles produced by coaxial EHDA led to slower release of cisplatin compared with the uniform particles. On the basis of these previous findings, two different types of CS structures were prepared with either both drugs in the core (CS1-cis-dac-PLGA) or cisplatin in the core and decitabine in the shell (CS2-cis-PLGA-dac). In order to evaluate drug–drug and drug–polymer interactions, CS NPs were compared with uniform NPs (U-cis-dac-PLGA), in which both drugs were dispersed throughout the polymer matrix.

**Fig. 2:**
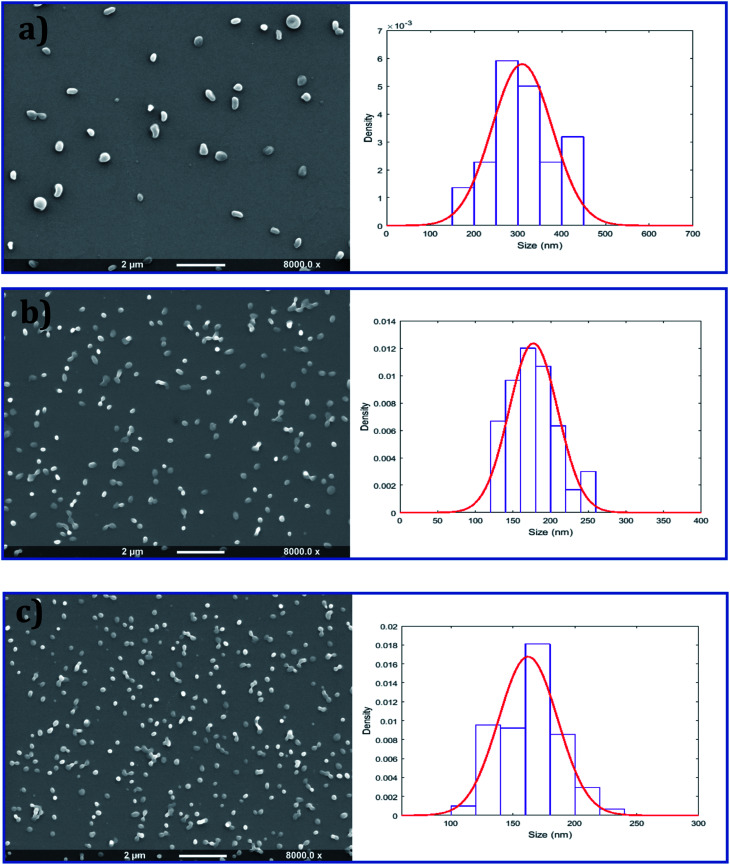
Scanning electron microscope images and corresponding size distribution graphs of (a) U-cis-dac-PLGA, (b) CS1-cis-dac-PLGA and (c) CS2-cis-PLGA-dac NPs produced by single needle (a) and coaxial (b) and (c) EHDA configurations.

**Fig. 3:**
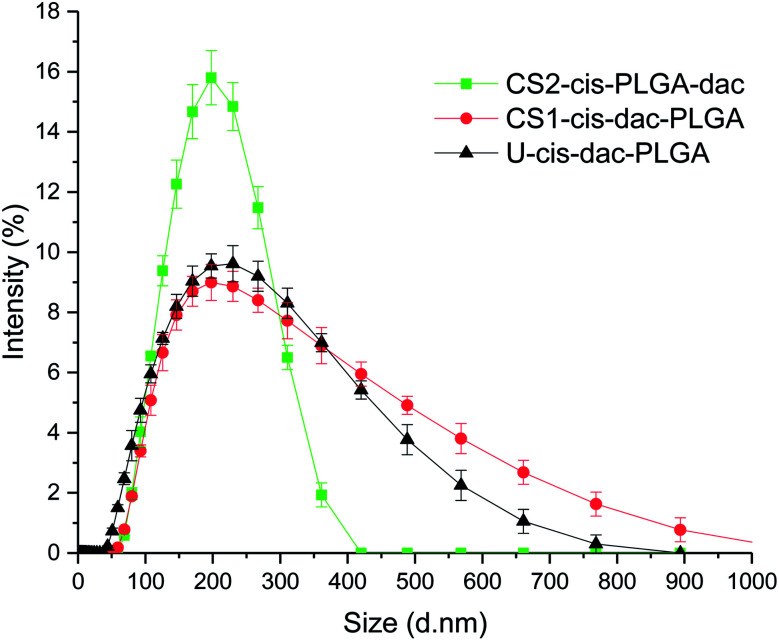
Size distribution profiles obtained by dynamic light scattering measurements for U-cis-dac-PLGA, CS1-cis-dac-PLGA and CS2-cis-PLGA-dac NPs.

As shown in Fig. 2, all three types of NP were spherical and had a smooth outer surface with no pores observed in the SEM images. The size distribution of the NPs was determined by measuring the diameter of 300 particles randomly chosen from each sample. Uniform particles produced by single needle EHDA had the largest mean diameter of 310 ± 68 nm, coupled with high encapsulation efficiencies (>80% for cisplatin and >70% for decitabine). When both drugs were encapsulated in the core through coaxial needle EHDA, CS1-cis-dac-PLGA NPs with mean diameter of 178 ± 34 nm were produced. The second group of CS particles with drugs in different layers, CS2-cis-PLGA-dac NPs, gave the smallest mean diameter of 162 ± 24 nm. The encapsulation efficiencies were slightly reduced in this case (*ca.* 70% and 50% for cisplatin and decitabine respectively). The slight increase in loss of decitabine could be attributable to its greater solubility. The *Z*-average size distribution and polydispersity index (Pdl) values of the samples were also measured using dynamic light scattering (DLS). All samples were found to have a high homogeneity and narrow size distribution, as shown in Fig. 3. The *Z*-average values were found to be as following: 276.9 ± 2.8 nm (Pdl = 0.54 ± 0.07) for U-cis-dac-PLGA NPs; 242.2 ± 2.5 nm (Pdl = 0.43 ± 0.12) for CS1-cis-dac-PLGA NPs; and finally 161 ± 3.7 nm (Pdl = 0.12 ± 0.02) for CS2-cis-PLGA-dac NPs.

#### Electron microscopy

**Fig. 4:**
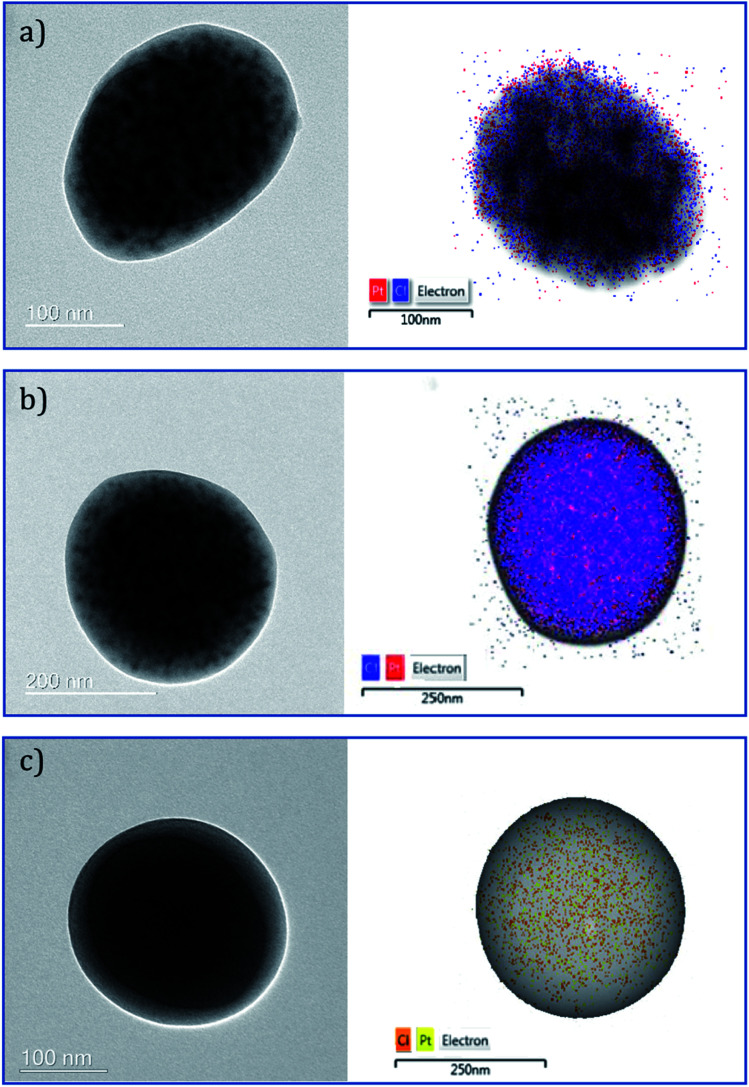
TEM bright-field and STEM micrographs of (a) U-cis-dac-PLGA, (b) CS1-cis-dac-PLGA and (c) CS2-cis-PLGA-dac NPs with overlaid EDS Pt (red for (a) and (b)), (yellow for (c)) and Cl mapping (blue for (a) and (b)), (amber for (c)).

In order to further investigate the encapsulation and distribution of cisplatin in the PLGA NPs, TEM analysis was performed. Micrographs of all three groups of NPs showed areas of darker intensity with the presence of cisplatin. The distribution of cisplatin within all three types of NPs was evaluated using energy-dispersive X-ray spectroscopy (EDS) Pt mapping. It can be suggested that cisplatin is well distributed within the polymer matrix in the case of the single needle derived NPs (Fig. 4). Consistent with previous observations,^33^ the distribution of cisplatin appears to be more concentrated in the core of the particles in both the coaxial derived CS PLGA particles. The variation in intensity can be observed in the TEM micrographs confirming a CS structure (Fig. 4b and c).

#### Fourier transform infrared spectroscopy

**Fig. 5:**
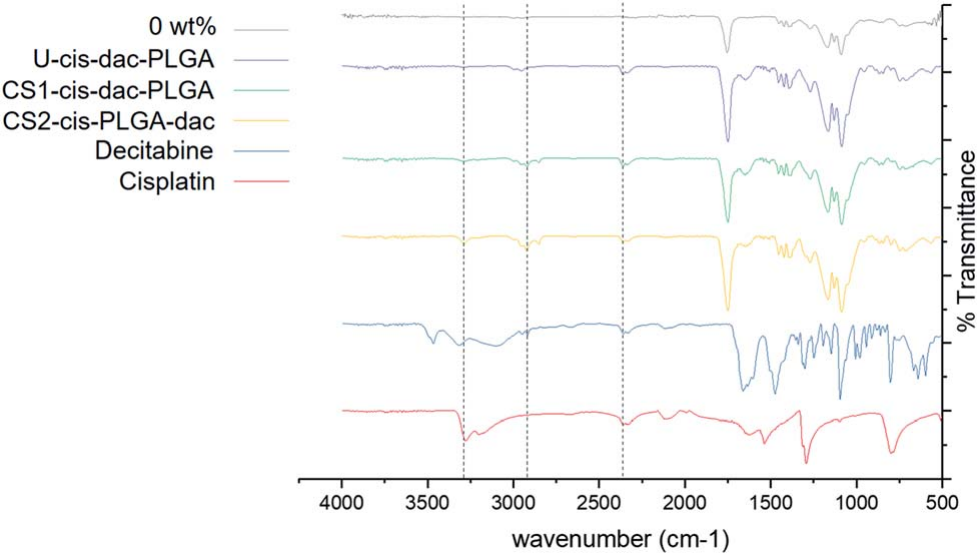
FTIR spectra of: cisplatin, decitabine and PLGA, U-cis-dac-PLGA, CS1-cis-dac-PLGA and CS2-cis-PLGA-dac particles.

The interaction between the drugs and PLGA following encapsulation were further examined by FTIR. A representative spectrum of all three NP samples with both drugs and unloaded PLGA particles as well as pure drugs is shown in Fig. 5. Pure cisplatin exhibited characteristic peaks including asymmetric and symmetric stretching of NH group (3274 cm^−1^), HNH asymmetric and symmetric bending (1300–1600 cm^−1^) region and around 800 cm^−1^ (related to HNH in plane bending). The pure decitabine sample showed characteristic peaks such as C–H stretching (alkane) at 2918 cm^−1^ and stretching of NH group (3467 cm^−1^). All the NPs demonstrated characteristic PLGA peaks that attribute to carbonyl C=O stretching (1754 cm^−1^) and C–O stretching of carboxylic acid (1050–1250 cm^−1^), a weak peak for amine stretching (3274 cm^−1^), indicating the presence of intact cisplatin and a peak for C–H stretching around 2850–2960 cm^−1^, confirming no interaction between the drug or polymer molecules. In addition, no peaks corresponding to cisplatin and decitabine were present in the unloaded PLGA particles.

### *In vitro* drug release characteristics

**Fig. 6:**
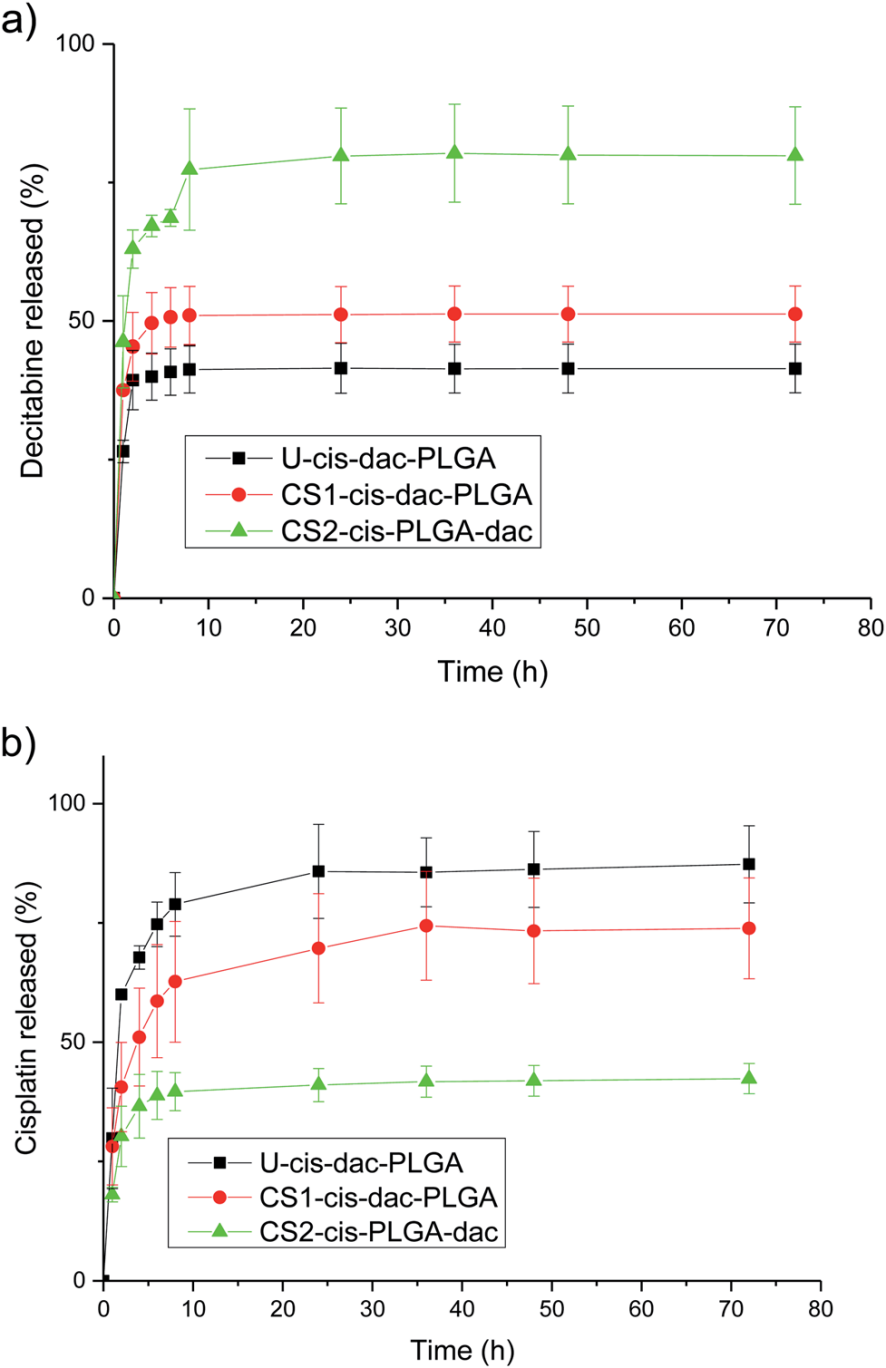
Structure dependent release for (a) decitabine and (b) cisplatin. PLGA dense CS particles were incubated in PBS at 37 °C and pH 7.4. The data show the mean ± SD from *n* = 3 samples.

To evaluate the influence of the different drug distribution and composition of the NPs on their release kinetics, *in vitro* release measurements of decitabine and cisplatin were performed (Fig. 6). Decitabine is a hydrophilic drug with a short half-life (25 minutes) and acts by hypomethylating DNA, making tumour cells less resistant to cisplatin.^34^ As a result, it is desirable for decitabine to be released at a faster rate than cisplatin. A significant burst release of decitabine was observed in the case of CS2-cis-PLGA-dac (Fig. 6a). Approximately 68% of decitabine was released in <5 h, while the remaining drug was released at a slower rate. In comparison, for the case of U-cis-dac-PLGA and CS1-cis-dac-PLGA NPs, slower release profiles were observed with 40% and 50% of the drug released in <5 h, respectively. The burst release phenomenon can be attributed to two main factors. First, drug molecules that are either loosely associated with the surface or embedded in the surface layer; and second, the pores and cracks associated with the polymeric particle morphology. In this case, an initial sharp release may be advantageous for targeted delivery and rapid treatment by decitabine prior to cisplatin release. In all three formulations decitabine was released faster than cisplatin. Therefore, it is important to select the formulation which gives the slowest cisplatin burst release to avoid side effects due to premature delivery of highly toxic cisplatin.^24^ As expected, U-cis-dac-PLGA NPs exhibited the highest cisplatin burst release with *ca.* 70% of cisplatin released within 5 h. In comparison, both the CS structure NPs demonstrated slower cisplatin release, with 37% cisplatin released from CS2-cis-PLGA-dac NPs and 55% from CS1-cis-dac-PLGA NPs in 5 h. The analysis of TEM/EDS (Fig. 4) shows the CS2-cis-PLGA-dac NPs have a region of higher cisplatin density with the core region of the NPs, hence the slowest release rate can be explained by the different architecture of the CS2-cis-PLGA-dac NPs.

In order to further analyse the mechanism of cisplatin and decitabine release, the release data were fitted to the analytical models described in the previous section. To investigate the sensitivity of the interpretation of the results to the CS structure, two studies were undertaken: in one case, the release profiles were fit assuming an initially uniform distribution of the drugs throughout the particles. In the other case the CS distribution was taken into account. For the uniform distribution model, the details of the structure were ignored and the release rates were fit using eqn (2) throughout. On the other hand, for the CS model, the release rates were fitted using eqn (2)–(4) as appropriate. Diffusion coefficients were obtained for all three formulations of U-cis-dac-PLGA, CS1-cis-dac-PLGA and CS2-cis-PLGA-dac (ESI, Tables A1 and A2†).

The changes in the diffusion coefficients according to the model used follow the expected trends (Tables A1 and A2†). For example, when the CS model is used the diffusion coefficient (4.4 × 10^−19^ m^2^ s^−1^ for U-cis-dac-PLGA, 2.9 × 10^−19^ m^2^ s^−1^ for CS1-cis-dac-PLGA and 2 × 10^−20^ m^2^ s^−1^ for CS2-cis-PLGA-dac), for decitabine is almost the same as that deduced from the uniform model (4.4 × 10^−19^ m^2^ s^−1^ for U-cis-dac-PLGA, 1.8 × 10^−19^ m^2^ s^−1^ for CS1-cis-dac-PLGA and 7 × 10^−20^ m^2^ s^−1^ for CS2-cis-PLGA-dac), as it is contained in the surface shell. Whereas the diffusion coefficient for cisplatin is increased to account for the fact that it has to penetrate the outer shell before it can begin to be released. These changes arising from the modelling assumptions, however, are small compared with the range of values obtained for decitabine, where the deduced diffusion coefficient differs by a factor of ∼20 for the different formulations. For cisplatin, by contrast, the variation is less than a factor of 2. This, combined with the different limiting release fractions for the different formulations, suggests that there might be structural differences on the nanoscale which have not been included in the model. This correlates with the variation in decitabine loading efficacy. Differences in chemical structure have been shown to influence the degradation and release rate of drugs from PLGA matrices.^35^ Therefore, it can be hypothesised that the molecular structure of the drug as well as the particle architecture are key in controlling drug release in this case.

### *In vitro* efficacy of nanoparticles

The NP formulation is designed to act as an intracellular delivery agent for increased cisplatin and decitabine efficacy. It is necessary to reduce premature release which both reduces drug efficacy and increases systemic toxicity. Previously, the authors have demonstrated that cisplatin efficacy could be increased *in vitro* by encapsulation within NPs and engineering the localisation of drug within the particle architecture.^33^ When using a dual drug system in the treatment of drug resistant cancers, temporal control over the relative release of individual drugs is also highly desirable, particularly if a ‘pre-treatment’ type of dosing may alter efficacy (as in the case of decitabine and cisplatin).^10^ Therefore, it is crucial to evaluate the effect of encapsulation and dual agent delivery against resistant cancer cells, with the aim of enhancing chemotherapy.

### *In vitro* anticancer activity of cisplatin loaded nanoparticles

**Fig. 7:**
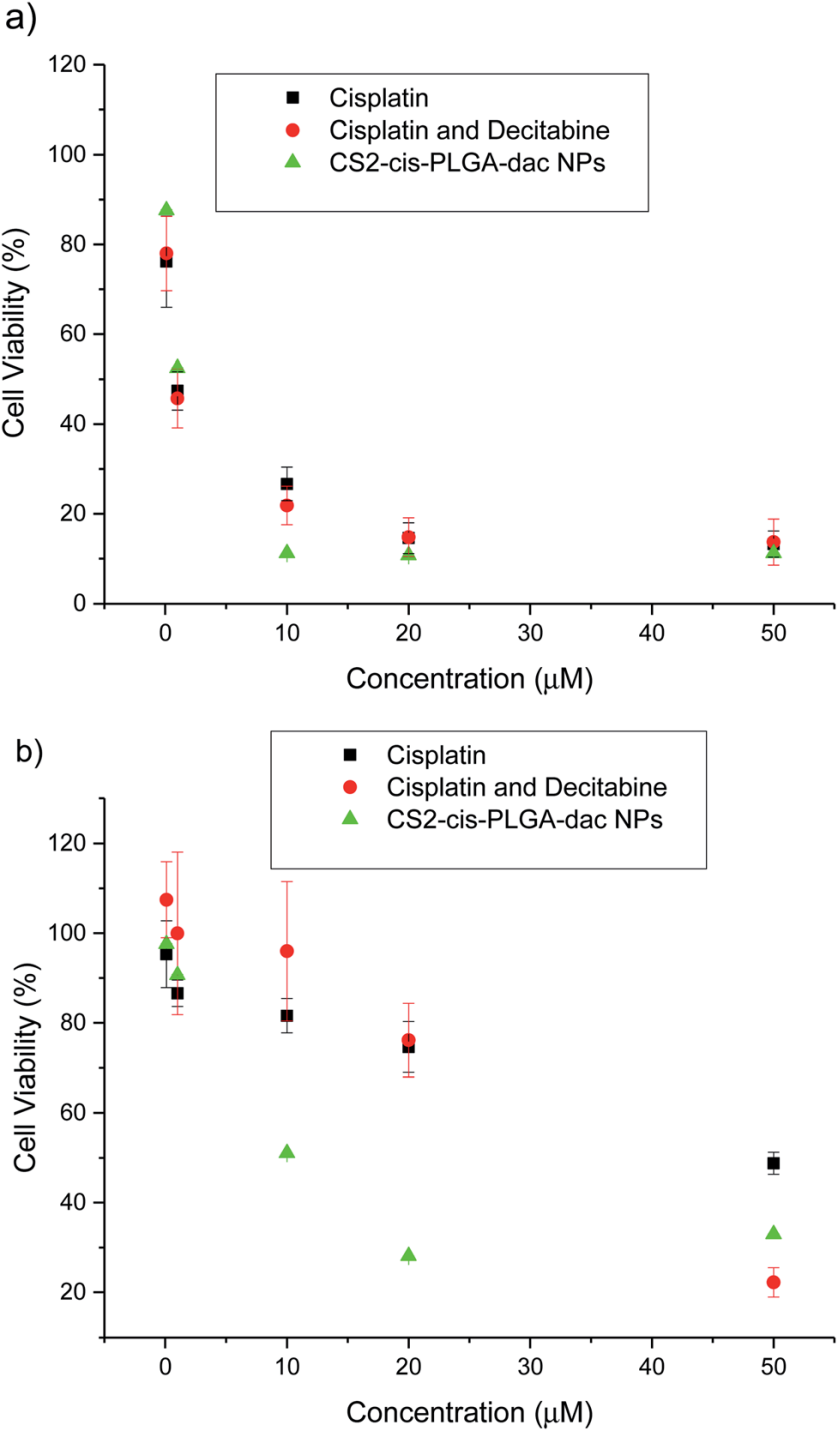
*In vitro* cytotoxicity of free cisplatin, combined free cisplatin and decitabine, and CS2-cis-PLGA-dac NPs against (a) non-resistant and (b) resistant A2780 cells after incubation for 72 h. The results represent the mean ± SD (*n* = 6).

**Fig. 8:**
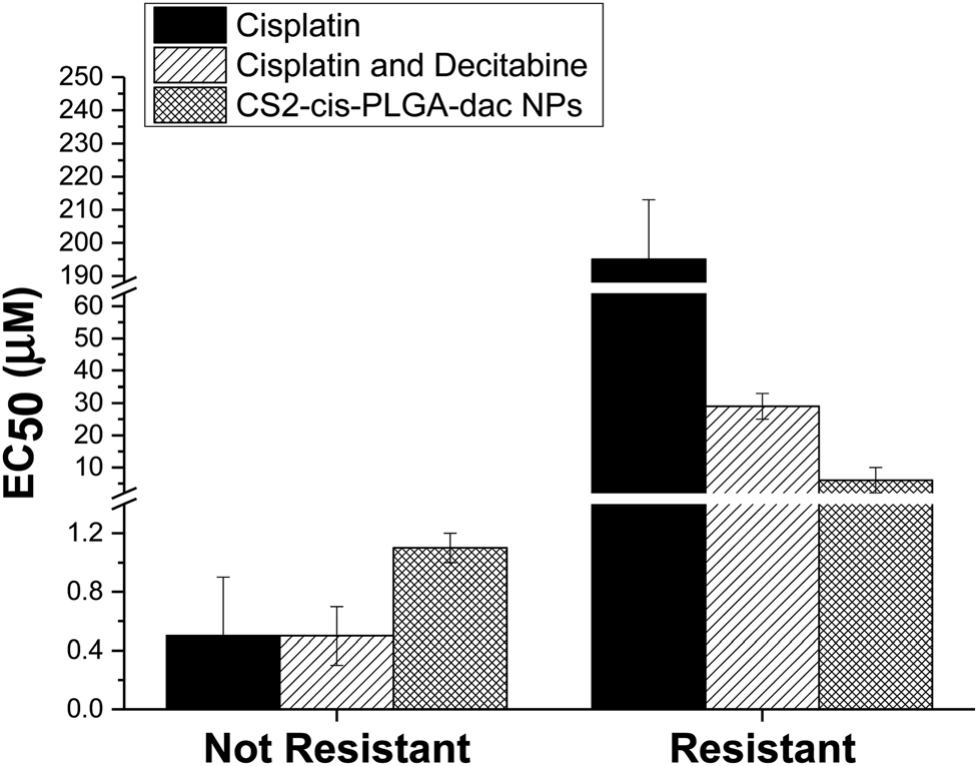
EC50 values for free cisplatin, combined free cisplatin and decitabine, and CS2-cis-PLGA-dac NPs after incubation for 72 h. The results represent the mean ± SD (*n* = 6).

The cytotoxic effect of free cisplatin, free dual drug treatment (cisplatin and decitabine) and dual drug loaded (CS2-cis-PLGA-dac) NPs was evaluated *in vitro*, using both resistant and non-resistant human ovarian carcinoma A2780 cell lines (Fig. 7). The dual drug loaded (CS2-cis-PLGA-dac) NPs demonstrated cytotoxicity against both resistant and non-resistant cell lines, suggesting effective delivery of both cisplatin and decitabine. In the case of the non-resistant cell line, the CS2-cis-PLGA-dac NPs generally exhibited an *in vitro* anticancer activity similar to when cisplatin was combined with decitabine as well as the free drug treatment at the same dosages. For example, in non-resistant cell lines, at a dosing of 10 μM free cisplatin caused a reduction in cell viability to 27%, in comparison to 22% and 11% for cisplatin and decitabine free drug and dual drug NPs respectively. As the cells are not resistant to cisplatin, both free drug and the combination treatment have shown to be more effective than the CS2-cis-PLGA-dac NPs. This is evident from the EC_50_ values recorded in Fig. 8 for both cases (EC_50_ = 0.5 μM) compared to CS2-cis-PLGA-dac NPs (EC_50_ = 1.1 μM). This can be explained by the prolonged release of cisplatin from CS2-cis-PLGA-dac NPs at 72 h. Therefore, the actual dosage given compared to the free drug is comparatively lower. Importantly, large differences in cell viability were observed between drug treatments for the cisplatin resistant cell line. Low levels of cytotoxicity were observed for this cell line when using cisplatin alone (EC_50_ = 195 μM), indicative of its resistant status (Fig. 8). In contrast, the cytotoxicity observed from the dual drug treatment was much greater, with EC_50_ values of 29 and 6 μM for free drugs and CS2-cis-PLGA-dac NPs, respectively.

It has been reported that cisplatin resistance could be reversible using decitabine, through increased sensitivity.^10^ One of the mechanisms for cisplatin resistance is DNA methylation which is an epigenetic silencing mechanism and can be reversible by demethylating drugs such as decitabine. Our results are in agreement with these findings, furthermore, the greater cytotoxicity demonstrated by the CS NPs in comparison to dual drug alone suggests further enhancements in efficiency due the delivery system. Within the experimental timeframe (72 h) around 70% of the decitabine from the shell of the CS2-cis-PLGA-dac NP formulation had already been released, allowing for successful cisplatin sensitisation of the cells. In contrast, only ≈40% of cisplatin had been released, yet importantly, cell viability was lower for the NP formulation compared to free drug. Recently, it was demonstrated that CS PLGA NPs containing cisplatin enabled sustained drug release and effective cellular internalisation, leading to enhancements in cytotoxicity.^33^ More effective intracellular delivery and the extended exposure period afforded by the NPs can similarly explain the increase in cytotoxicity in non-resistant cells observed in this study.

## Conclusions

Co-encapsulation of decitabine and cisplatin in a single nanocarrier was achieved by EHDA. This technique enabled different NP structures to be generated and, as a result, the drug release characteristics to be tuned. The co-encapsulated formulation offering the most desirable release profile, with rapid decitabine release and slower cisplatin release was then tested to determine its cytotoxicity in normal and cisplatin resistant tumour cells. Compared with free cisplatin and free cisplatin and decitabine, the NPs exhibited higher cytotoxicity in resistant cancer cells after incubation for 72 h. This broad adaptability of the CS NP enables the delivery of potentially synergistic drug ratios to achieve the maximum therapeutic effect. CS NPs can be used as a potential therapeutic tool to overcome the chemoresistance of cisplatin.

## Conflicts of interest

There are no conflicts to declare.

## Supplementary Material

NA-002-C9NA00684B-s001
