# Supplementary material for: Enhanced efficacy in drug-resistant cancer cells through synergistic nanoparticle mediated delivery of cisplatin and decitabine
Source: Nanoscale Adv. 2020 Jan 27;2(3):1177–86. doi: 10.1039/c9na00684b (PMC9419023; doi:10.1039/c9na00684b)
Supplement: NA-002-C9NA00684B-s001 [file NA-002-C9NA00684B-s001.pdf]

Electronic Supplementary Material (ESI) for Nanoscale Advances.
This journal is © The Royal Society of Chemistry 2020

Supporting Information

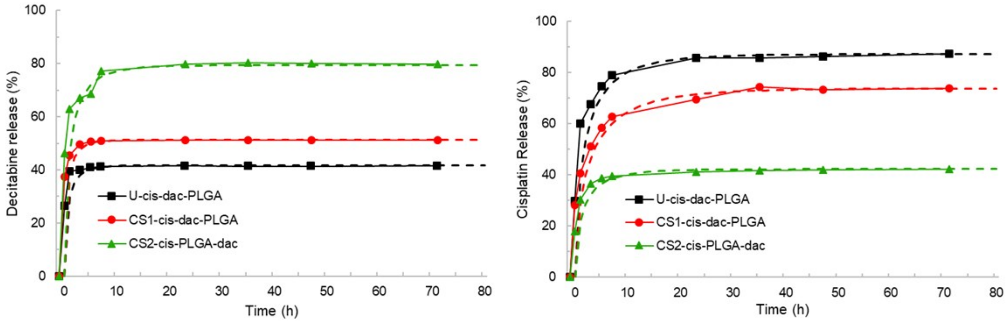

Figure A1. Comparison of experimental release rates (points and dashed lines) with the fitted model neglecting the CS structure of the particles (uniform model).

Formulation	Drug	*D* m^2s^{-1}
U-cis-dac-PLGA	Cisplatin	1.4×10^{-19}
CS1-cis-dac-PLGA	Cisplatin	4×10^{-20}
CS2-cis-PLGA-dac	Cisplatin	5×10^{-20}
U-cis-dac-PLGA	Decitabine	4.4×10^{-19}
CS1-cis-dac-PLGA	Decitabine	1.8×10^{-19}
CS2-cis-PLGA-dac	Decitabine	7×10^{-20}

Table A1: Diffusion coefficients obtained using the uniform sphere model.

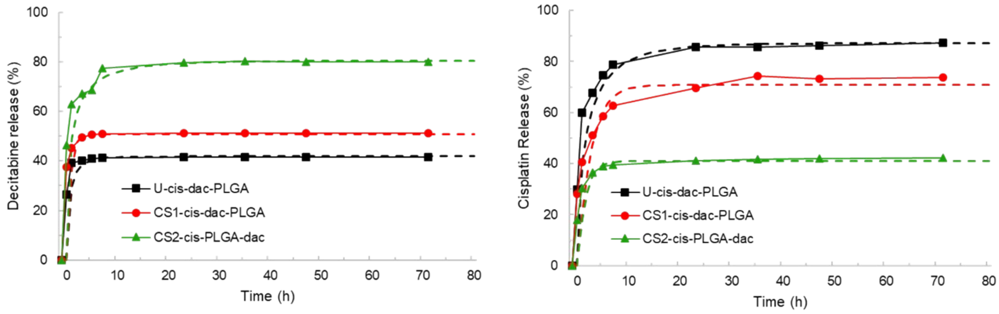

Figure A2. Comparison of experimental release rates (points and dashed lines) with the fitted model taking account of the CS structure of the particles (CS model).

Formulation	Drug	*D* m^2s^{-1}
U-cis-dac-PLGA	Cisplatin	1.4×10^{-19}
CS1-cis-dac-PLGA	Cisplatin	8×10^{-20}
CS2-cis-PLGA-dac	Cisplatin	1.1×10^{-19}
U-cis-dac-PLGA	Decitabine	4.4×10^{-19}
CS1-cis-dac-PLGA	Decitabine	2.9×10^{-19}
CS2-cis-PLGA-dac	Decitabine	2×10^{-20}

Table A2: Diffusion coefficients obtained using the CS model.

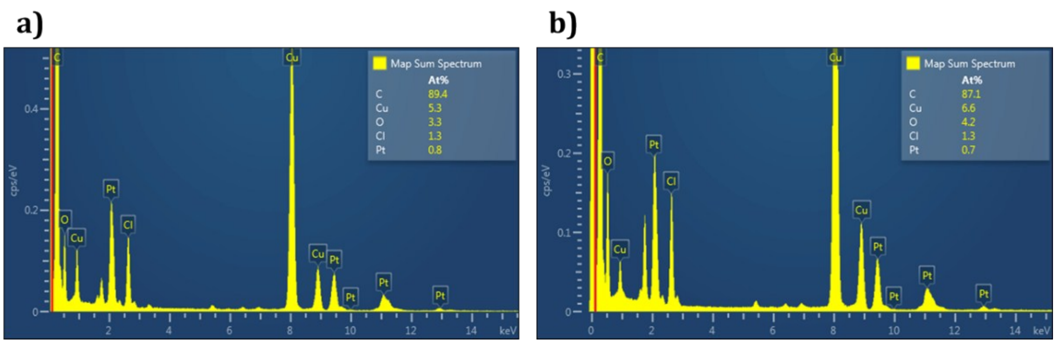

Figure A3. Typical EDS spectra of cisplatin region of a) CS2-cis-dac-PLGA and b) CS2-cis-PLGA-dac
